# Consecutive soybean (*Glycine max*) planting and covering improve acidified tea garden soil

**DOI:** 10.1371/journal.pone.0254502

**Published:** 2021-07-13

**Authors:** Shuilian Gao, Peng He, Tianxiu Lin, Haijuan Liu, Bin Guo, Huiling Lin, Yunfei Hu, Qianjie Chen, Ping Xiang, Lifeng Zou, Xinghui Li, Zhongguo Xiong, Jinke Lin

**Affiliations:** 1 Anxi College of Tea Science, Fujian Agriculture and Forestry University, Quanzhou, Fujian, China; 2 The College of Horticulture, Fujian Agriculture and Forestry University, Fuzhou, Fujian, China; 3 Tea Research Institute, Nanjing Agriculture University, Nanjing, Jiangsu, China; 4 School of Plant Sciences, BIO5 and College of Agriculture and Life Sciences, University of Arizona, Tucson, Arizona, United States of America; University of Palermo, ITALY

## Abstract

Planting soybeans (*Glycine max* (L.) Merr.) in tea gardens decreased soil pH in theory but increased it in practice. This controversy was addressed in this study by treating the tea garden soil consecutively with different parts of a soybean cover crop: aboveground soybean (ASB) parts, underground soybean (USB) root residues, and the whole soybean (WSB) plants. In comparison with the control, the soil pH increased significantly after the third ASB and WSB treatments, but there was no significant change in the soil pH in the USB treatment. Concordantly, the soil exchangeable acidity decreased significantly and the soil exchangeable bases increased significantly in the ASB and WSB treatments. The exchangeable acidity increased in the USB treatment, but the amount of the increased acidity was less than that of the increased bases in the ASB treatment, resulting in a net increase in the exchangeable bases in the WSB treatment. Soybean planting and covering also increased the microbial richness and abundance significantly, which led to significantly more soil organic matters. Exchangeable K^+^ and Mg^2+^, and soil organic matters played significantly positive roles and exchangeable Al^3+^ played negative roles in improving soil pH. Our data suggest that consecutive plantings of soybean cover crop increase the pH of the acidified tea garden soil.

## Introduction

Tea plants (*Camellia sinensis* (L.) O. Ktze.) grow well in acidic soil with an optimum pH between 4.5 and 5.5 [1], but the tea garden soil has been over-acidified in general. More than 46.0% of Chinese tea gardens were reported to have a soil pH < 4.5 [2] and more than 38.64% of Japanese tea gardens were reported to have a soil pH < 4.0 [3]. The severe acidification can not only harm the soil ecology and retard the growth of tea plants [4], but also lead to accumulation of toxic metal compounds in tea plants and leaves, potentially creating a safety risk for the tea industry [5].

Planting leguminous crops is an effective way to improve soil by providing biologically fixed nitrogen (N) and enriching beneficial bacterial communities [6, 7], but its effect on the soil pH has been controversial. In theory, legume root systems are expected to accelerate soil acidification. The rhizobial community in the root system fixes N to NH_3_, and subsequent production of NH_4_^+^ by mineralization leads to nitrification or hydrolysis reaction that releases H^+^ [8, 9]. Accumulation of NH_4_^+^ can also increase the content of exchangeable Al^3+^ in soil, consequently accelerating soil acidification [10]. However, many practices of intercropping soybean (*Glycine max* (L.) Merr.) or other legumes in tea gardens have increased the soil pH [11, 12]. This controversy may be caused by the differential effects of different soybean plant parts. The underground soybean (USB) root system may accelerate acidification of tea garden soil, but the aboveground soybean (ASB) parts may increase the soil pH because humus degraded by microbes from the ASB organic matters adsorbs H^+^ and exchanges out OH^-^ [13].

To examine the controversy that planting soybeans in tea gardens decreases soil pH in theory but increases it in practice, this study dissected the effects of different parts of soybean plants on the soil pH and microbial abundance in a highly acidified tea garden soil (pH 4.36). Soybeans were planted in acidified tea garden soil in pots. Residues of ASB, USB, and the whole soybean (WSB) plants were left in soil or as soil cover after harvest. Soil pH and exchangeable cations were monitored over two consecutive years. N forms, organic matter, and bacterial and fungal abundances were determined after three consecutive applications of the soybean planting and covering.

## Materials and methods

### Ethics statement

No permit was required for soil sampling in the field as the samples were taken from the tea garden of a private company, Fujian Niannianxiang Tea Co., Ltd., China. The plan to sample the soils (locations and volumes) was explicitly approved by the owner of the company, Mr. Tiande Li. There was no protected species sampled in this study.

### Soil used

Ferralic Nitisol (red loam) soil was collected from a tea garden with a history of 15 consecutive years of tea planting. Five samples of top soil were taken from a depth of 0–30 cm between the rows of tea plants after removing the surface debris. After thorough mixing, 18 kgs of soil was placed in each large plastic pot (27 cm x 33 cm, H x D). The pH of the mixed soil was measured at 4.36±0.01, the organic matter content at 1.36±0.06%, and exchangeable K^+^, Na^+^, Ca^2+^, Mg^2+^, Al^3+^, H^+^ contents at 0.95±0.04, 1.27±0.03, 6.18±0.08, 0.26±0.02, 2.14± 0.05, 0.48±0.03 cmol/kg, respectively.

### Soybean cultivation

Twelve seeds of soybean cultivar Huaxia-1 were planted successively on April 1, 2017; July 1, 2017; and May 7, 2018 in each pot outdoors. At this location (118°13′50″E, 25°4′45″N), the annual average temperature is 20°C, and the annual rainfall is 1600 mm. During the first two plantings, 42.5 g/pot of fused calcium-magnesium phosphate fertilizer (15% of P_2_O_5_, 45% of CaO, 20% of SiO_2_, 12% of MgO, and 8% of impurities) was applied once before planting as the base fertilizer. Each pot was irrigated with 600 ml of tap water every 5 arid days.

### Experimental design

Three treatments, ASB, USB, and WSB, were set up at the early seed-filling stage of soybean plants. In ASB, the aboveground parts of soybean plant cut from the USB treatment were used to cover the soil in pots with no prior soybean planting. In USB, the aboveground parts of the soybeans were removed and only the underground roots of soybean plants remained in the soil. In WSB, the above ground parts of soybean plants were cut but left as soil cover in the same pot, and the underground roots remained undisturbed. Each treatment consisted of 8 replicates. Additionally, 4 replicates of no soybean planting were set up as a blank control block (CK). All treatments, including CK, were fertilized and irrigated exactly the same. Three successive ASB, USB, and WSB treatments were carried out on July 1, 2017; September 9, 2017; and August 1, 2018 after the corresponding crop of soybean plants reached the early seed-filling stage. One random soybean plant from each pot was chosen for the dry weight measurements of the whole, the aboveground parts, and the root system of soybean plants. Root nodules were removed and measured before plants were dried in a hot air oven at 105°C. Plant residues and the removed root nodules were returned to their original pots after the measurements.

### Soil sampling and analysis

Soil samples were collected on September 9, 2017, May 7, 2018, December 31, 2018 after the completion of the 1st, 2nd, and 3rd applications of each treatment, respectively, when the decomposition of the soybean residues was nearly completed. Plant residues were removed before vertical soil samples of about 60 g were taken from a depth of 0 cm to 15 cm. Five random samples from each pot were mixed as a composite. Portions of the composite samples were immediately used for the measurements of the following soil characteristics: microbial populations (3 g soil), enzymatic activities (30 g soil), and different forms of N (10 g soil), while the remaining soil was air dried and used for the measurements of the soil pH (20 g soil), exchangeable cations (14 g soil), and organic matters (1 g soil).

The following methods were used to measure different soil characteristics [14]: pH by potentiometry, exchangeable K^+^ and Na^+^ by ammonium acetate exchange-flame photometry, exchangeable Ca^2+^ and Mg^2+^ by ammonium acetate exchange-atomic absorption spectrophotometry, exchangeable Al^3+^ and H^+^ by KCl exchange-neutralization titration, and soil organic matter by the K_2_Cr_2_O_7_ oxidation-volume method with external heat. NH_4_^+^ and NO_3_^-^ were measured with a KCL extraction-flow analyzer by the Nanjing Soil Research Institute, China. Each sample was measured twice for accuracy.

The soil microorganism population was determined by using the next generation sequencing (NGS) analysis of the V3-V4 region of bacterial 16S rDNA and fungal ITS regions by Biomarker Technologies Corporation, Beijing, China [15]. Briefly, 6 independent soil samples were taken from each treatment and 3 independent soil samples were taken from CK. Total DNA extracted from each sample with the NucleoSpin 96 Soil DNA kit (Macherey-Nagel, Germany) was used as templates for the library preparations of bacterial 16S rDNA and fungal ITS region. Bacterial rDNA was initially amplified with the primers 338F 5’-ACTCCTACGGGAGGCAGCA-3’ and 806R 5’-GGACTACHVGGGTWTCTAAT-3’ and the fungal ITS DNA was initially amplified with primers ITS1F (5’-CTTGGTCATTTAGAGGAAGTAA-3’) and ITS2 (5’-GCTGCGTTCTTCATCGATGC-3’) for the preparation of the libraries. Paired-end sequencing of the libraries was performed on the Illumina HiSeq 2500 platform. Paired sequencing reads with a minimum overlap of 10 bases and a maximum mismatch rate of 0.2 were merged as raw tags using FLASH v1.2.11 [16]. The raw tags were then filtered and cleaned using Trimmomatic v0.33 [17] and UCHIME v8.1 [18] to obtain clean and valid tags. Clean tags with 97% sequence identity was clustered together as an operational taxonomic unit (OTU) using USEARCH v10.0 [19]. OTUs were classified according to the SILVA ribosomal RNA gene database for bacterial and archaeal species [20] and to the UNITE database for the molecular identification of fungi [21]. The richness of soil microbiomes were measured by both the ACE (abundance-based coverage estimator) index and the Chao1 index (number of expected OTUs in a sample among all OTUs identified in all samples) [22].

Soil urease activities were determined using the sodium phenoxide-sodium hypochlorite method [23], sucrose activities were determined by the 3,5-dinitrosalicylic acid method [24], and nitrate reductase activities were determined using the phenol disulfonic acid method [25].

### Data analysis

Descriptive statistics were generated by SPASS v17.0 to remove outliers with absolute standard deviation >3. The removed data were one entry each of exchangeable Ca^2+^ in the first CK, USB, WSB treatments; two and one entry of exchangeable Mg^2+^ in the second ASB and WSB treatment, respectively; one entry of exchangeable Al^3+^ in the third WSB treatment and one entry of exchangeable Na^+^ in the third USB treatment; one entry each of NH_4_^+^ in CK and WSB treatments; and one entry of NO_3_^-^ in USB treatment. ANOVA-Duncan multiple range tests were used to evaluate the significance of differences in means of pH, exchangeable cations, organic matter, and microbial abundance. The ACE and Chao1 indices of microbial abundances were computed from the OTUs identified from each treatment with Mothur 1.30 [26]. Redundancy analysis was performed in Canoco 5 [27] to identify soil chemical and physical factors influencing the soil pH.

## Results

### Changes of tea garden soil pH in soybean planting and covering treatments

**Fig 1:**
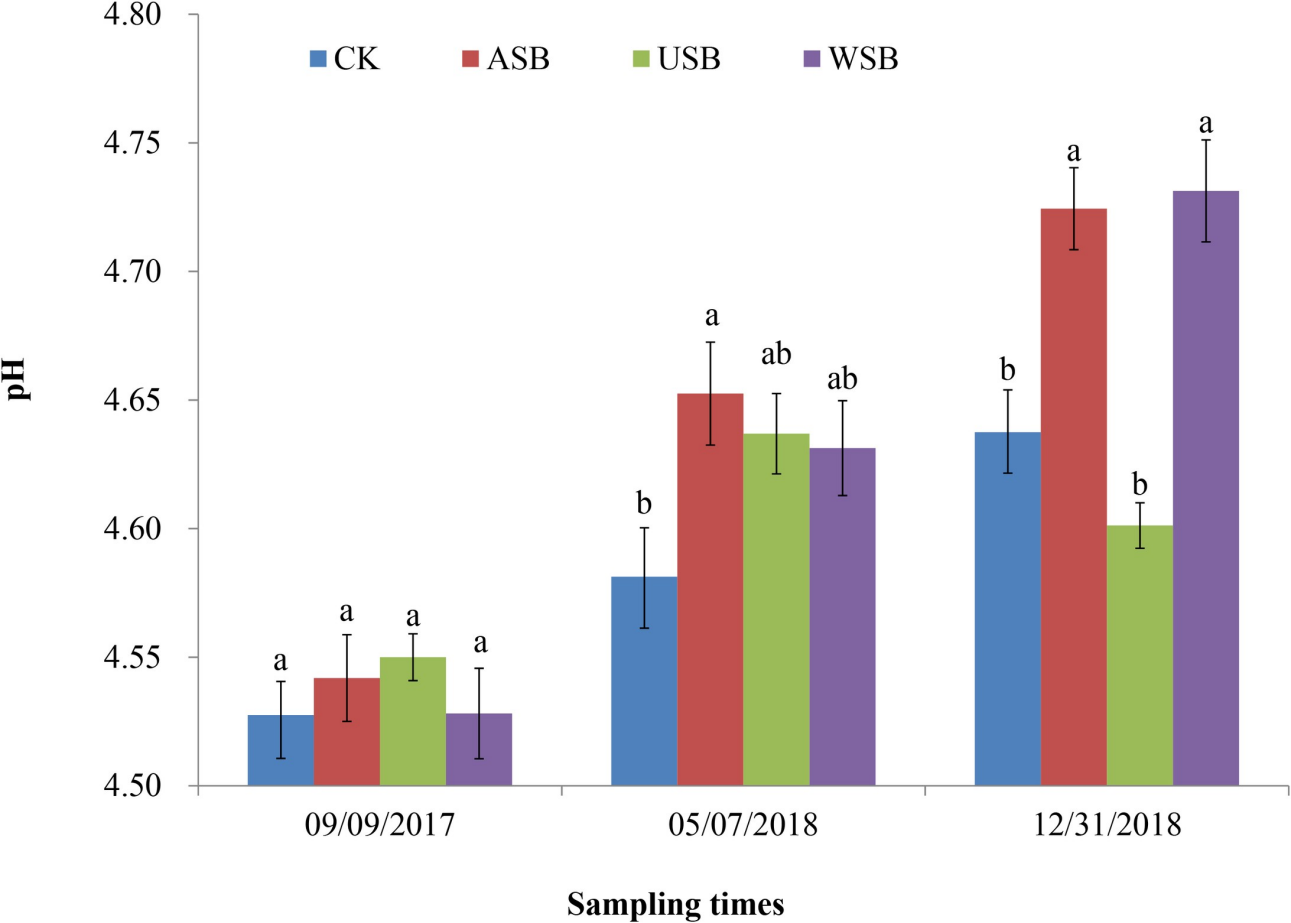
Changes of tea garden soil pH in soybean planting and covering treatments. Soil pH was measured at three time points after the completion of each application of various treatments. Error bar in each column represents ± standard error. Different lowercase letters denote significant differences among the means at the same time point as determined by the Duncan test (P<0.05). CK, control; ASB, aboveground soybean parts; USB, underground soybean parts; WSB, whole soybean plants.

To evaluate the overall effects of the ASB, USB, WSB, and CK treatments on the soil pH, soil samples were measured for pH at three time points after the completion of each application of the treatments (Fig 1, S1–S3, S6 Tables in S1 File). ANOVA analysis and Duncan multiple range tests of the data did not show a significant difference in the soil pH between different treatments after the first application. However, the soil pH values increased significantly (P<0.05) by 0.07 after the second ASB treatment, and increased significantly by 0.08 and 0.09 after the third ASB and WSB treatments, respectively, when compared to those of the CK treatment. In contrast, the soil pH decreased slightly after the third USB treatment, but the decrease was statistically insignificant. Results from these experiments indicated that the aboveground soybean parts were the main contributor to the increase in soil pH, as demonstrated by the significant increases of the soil pH in the ASB and WSB treatments, and insignificantly change in the soil pH in the USB treatment. These data showed that consecutive soybean planting and covering increased the pH of the tea garden soil.

### Changes of soil exchangeable acidity and exchangeable bases in soybean planting and covering treatments

After the first ASB, USB, and WSB treatments, acidic exchangeable cations (exchangeable acidity), consisting of Al^3+^ and H^+^, decreased slightly but insignificantly across all treatments in comparison with CK. Basic exchangeable cations (exchangeable bases), consisting of exchangeable K^+^, Na^+^, Ca^2+^ and Mg^2+^, decreased significantly (P<0.05) in the USB treatment in comparison with CK (Table 1, S1–S3 Tables in S1 File), but no insignificant changes were observed in the ASB and WSB treatments.

**Table 1:** Changes of soil exchangeable acidity and exchangeable bases in soybean planting and covering treatments.

Sampling dates	Exchangeable cations^a^	CK (cmol/kg)^b^	ASB (cmol/kg)	USB (cmol/kg)	WSB (cmol/kg)
09/09/2017	acidic	2.68±0.09a	2.68±0.05a	2.61±0.08a	2.65±0.11a
	basic	10.66±0.13a	10.52±0.12ab	10.18±0.13b	10.43±0.14ab
05/07/2018	acidic	2.55±0.12a	2.04±0.09b	2.35±0.06a	2.34±0.08a
	basic	10.46±0.12c	11.01±0.20ab	10.56±0.16bc	11.46±0.13a
12/31/2018	acidic	2.62±0.03a	2.40±0.06b	2.60±0.04a	2.11±0.08c
	basic	10.38±0.11c	11.58±0.13a	10.77±0.10b	11.93±0.14a

^a^ The exchangeable acidity and exchangeable bases were summed from exchangeable Al^3+^ & H^+^ and exchangeable K^+^, Na^+^, Ca^2+^ & Mg^2+^ in Table 2, respectively.

^b^ Data represent mean ± standard error. Different letters on the same line indicate significant differences among treatments as determined by the Duncan test (P<0.05). CK, control; ASB, aboveground soybean parts; USB, underground soybean parts; WSB, whole soybean plants.

**Table 2:** Exchangeable cations of tea garden soil in soybean planting and covering treatments.

Sampling dates	Exchangeable cation^a^	CK (cmol/kg)^b^	ASB (cmol/kg)	USB (cmol/kg)	WSB (cmol/kg)
09/09/2017	Al^3+^	2.27±0.09a	2.21±0.03a	2.21±0.07a	2.24±0.08a
	H^+^	0.41±0.01a	0.46±0.02a	0.41±0.03a	0.41±0.02a
	K^+^	0.90±0.06a	0.89±0.05a	0.64±0.05b	0.74±0.04b
	Na^+^	1.42±0.08a	1.09±0.10b	1.00±0.08b	1.13±0.10b
	Ca^2+^	8.05±0.02a	8.09±0.02a	8.08±0.02a	8.11±0.01a
	Mg^2+^	0.31±0.05b	0.45±0.02a	0.44±0.03a	0.44±0.03a
05/07/2018	Al^3+^	2.22±0.15a	1.68±0.08c	1.97±0.07b	1.93±0.07b
	H^+^	0.33±0.04b	0.36±0.03ab	0.38±0.02ab	0.41±0.01a
	K^+^	0.92±0.02b	1.55±0.12a	0.84±0.08b	1.73±0.07a
	Na^+^	1.09±0.05a	0.89±0.06a	1.10±0.08a	0.96±0.06a
	Ca^2+^	8.12±0.04a	8.23±0.08a	8.21±0.09a	8.31±0.08a
	Mg^2+^	0.32±0.04c	0.53±0.04a	0.40±0.02bc	0.49±0.03ab
12/31/2018	Al^3+^	2.34±0.03a	2.14±0.06b	2.42±0.05a	1.84±0.08c
	H^+^	0.28±0.02a	0.26±0.01a	0.18±0.01b	0.27±0.01a
	K^+^	0.49±0.04d	1.31±0.05b	0.69±0.05c	1.55±0.06a
	Na^+^	1.38±0.09a	1.24±0.04ab	1.28±0.05ab	1.12±0.05b
	Ca^2+^	8.30±0.06a	8.46±0.07a	8.44±0.04a	8.39±0.05a
	Mg^2+^	0.20±0.04d	0.57±0.05b	0.38±0.02c	0.86±0.04a

^a^ Soil exchangeable cations were measured after completion of each application of each treatment.

^b^ Data represent mean ± standard error. Different letters on the same line indicate significant differences among treatments as determined by the Duncan test (P<0.05). CK, control; ASB, aboveground soybean parts; USB, underground soybean parts; WSB, whole soybean plants.

After the second and third applications, the soil exchangeable acidity in the ASB treatment decreased by 20.00% and 8.40%, and the exchangeable bases increased by 5.26% and 11.56%, respectively in comparison with CK. All these changes were significant (P<0.05). Similar results from the WSB treatment were observed, with 8.24% and 19.47% decrease in the exchangeable acidity, and 9.56% and 14.93% increase in the exchangeable bases after the second and third applications, respectively. All these changes were significant (P<0.05) except the exchangeable acidity after the second WSB treatment. In the USB treatment, there was a slight decrease in exchangeable acidity and slight increases in exchangeable bases after all three applications. All of these changes were not significant (P<0.05) with the exception of the increase in the exchangeable bases after the third USB treatment (Table 1).

Since both the decrease in exchangeable acidity and the increase in exchangeable bases contributed to an increase of the soil pH, the sums of the absolute values of the decreased percentage of the exchangeable acidity and the increased percentage of the exchangeable bases could explain the level of the increases in soil pH. These sums were calculated as 25.26% and 19.96% in the ASB treatment, 17.80% and 34.40% in WSB treatment after the second and third applications of the treatments, respectively. These changes were much higher than 8.80% and 4.52% calculated for the USB treatment, indicating that the consecutive WSB and ASB treatments had accumulative effect in improving soil acidification by reducing the exchangeable acidity and increasing the exchangeable bases in soil.

### The roles of exchangeable cations played in controlling total soil exchangeable acidity and bases

When exchangeable Al^3+^, H^+^, K^+^, Na^+^, Ca^2+^, Mg^2+^ cations were measured and examined, the dominant roles of exchangeable Al^3+^, K^+^, Mg^2+^ cations in the change of the soil pH became apparent (Table 2, S1–S3 Tables in S1 File). The exchangeable Al^3+^ accounted for >82% in the exchangeable acidity in all treatments and its changes was positively correlated with those of the exchangeable acidity. Changes in exchangeable H^+^ were unstable, but the differences in most treatments were not significant (P<0.05), except significantly higher at the end of the second WSB treatment and significant lower at the end of third USB treatment. These data indicated that Al^3+^ played a dominant role in the exchangeable acidity.

Soil exchangeable K^+^ and Mg^2+^ increased rapidly after consecutive ASB and WSB treatments (Table 2, S1–S3 Tables in S1 File). In comparison with CK, exchangeable K^+^ increased by 68.48% and 167.35%, and exchangeable Mg^2+^ by 65.63% and 185.00% after the second and third ASB treatment, respectively. The exchangeable K^+^ increased by 88.04% and 216.33%, and exchangeable Mg^2+^ by 53.13% and 330.00% after the second and third WSB treatment, respectively. Even in the third USB treatment, both exchangeable K^+^ and Mg^2+^ increased significantly by 40.82% and 90.00%, respectively. The trend of these changes was consistent with those of the exchangeable bases, indicating that K^+^ and Mg^2+^ played dominant roles in the increase of the exchangeable bases.

The contents of exchangeable Ca^2+^ were consistently the largest component of the exchangeable bases in all the treatments, including the control, but there was no significant difference among the treatments. The original soil used in this study already contained a high level of exchangeable Ca^2+^ (6.18cmol/kg), and the additional Ca^2+^ might have been introduced by the fused calcium-magnesium phosphate fertilizer applied during the experiments. Changes in soil exchangeable Na^+^ were found to be irregular. No significant difference was found between treatments after the second treatments, but the exchangeable Na^+^ was significantly higher in CK and lower in the WSB treatment after the first and the third treatment, respectively.

### Soil NH_4_^+^ and NO_3_^-^ in soybean planting and covering treatments

**Fig 2:**
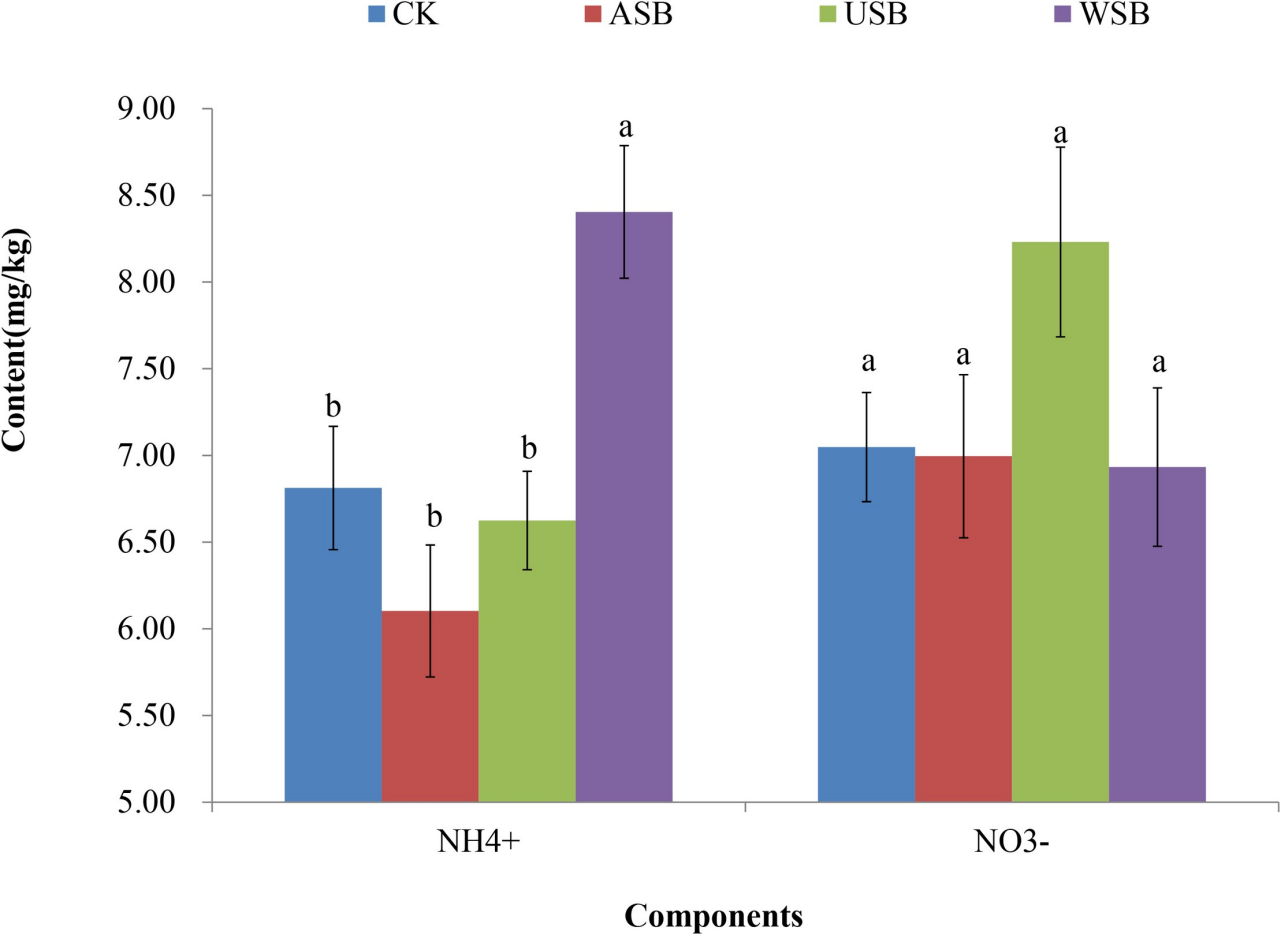
Contents of soil NHNH_4_^+^ and NO_3_^-^ in consecutive soybean planting and covering treatments. NH_4_^+^ and NO_3_^-^ were measured at the completion of the third application of the treatments. Error bar in each column represents ± standard error. Different lowercase letters indicate significant differences in the means of NH_4_^+^ or NO_3_^-^ among different treatments as determined by the Duncan test (P<0.05). CK, control; ASB, aboveground soybean parts; USB, underground soybean parts; WSB, whole soybean plants.

A major benefit of intercropping soybeans in tea gardens is the nitrogen fixed by rhizobial species in root nodules, which increases the availability of NH_4_^+^ in soil and improve soil fertility [28]. The average number of root nodules was 146±20 and the average fresh root nodule weight was 2.46±0.40g per pot in which soybeans were planted. Different forms of N were then measured after the third application of soybean planting and covering to determine nitrogen availability in soil (Fig 2, S4 and S7 Tables in S1 File). Soil NH_4_^+^ was significantly higher in the WSB treatment than in the other treatments while soil NO_3_^-^ was substantially but not significantly higher in the USB treatment than the other treatments, as determined by the Duncan test (P<0.05). These data indicated that the whole soybean plants increased soil NH_4_^+^ while soybean roots increased soil NO_3_^-^.

### The diversity of soil microbes and contents of soil organic matters in consecutive soybean planting and covering treatments

Soil microbes degrade soybean plant residues to form organic matter, humus. It is a key indicator of soil properties. Soil microbial communities were measured after the third application of the soybean planting and covering treatments. DNA from soil was extracted and analyzed by NGS sequencing of bacterial 16S rDNA and fungal ITS regions. After cleaning and filtering, a total of 1.29 million of high quality, clean, and unique tags were generated from the bacterial 16S rDNA sequencing for all the samples, with an average of 61,298±304 tags per sample and a minimum of 59,103 tags for the samples (S5 Table in S1 File). A total of 1.45 million unique clean tags were generated for the fungal ITS sequencing of all the samples, with an average of 69,085±1,129 tags per sample and a minimum of 51,660 tags for the samples (S5 Table in S1 File). Unique bacterial and fungal tags were then classified into operational taxonomic units (OTUs).

Soybean planting and covering had a significant effect on the richness of soil microbiomes, as measured by both the ACE index and the Chao1 index [22]. The results showed that soybean planting and covering (ASB, USB, and WSB treatments) significantly increased the microbial richness than the control (CK), more so in the fungal community than in the bacterial community (Table 3, S5 Table in S1 File). The richness of the bacterial community increased by 18%~26% while that of the fungal community increased by 42%~51%. The richness estimated by both ACE and Chao1 are comparable, confirming the reliability of the estimates. While the WSB and ASB treatments had a higher mean bacterial richness than the USB treatment, the ASB and USB treatment had a higher fungal abundance. However, these differences were not significant.

**Table 3:** Tea garden soil microbial richness in consecutive soybean planting and covering treatments.

Category	Abundance index	CK^a^	ASB	USB	WSB
Bacterium	ACE	1042.00±97.63b	1296.88±11.65a	1231.65±39.31a	1322.35±14.42a
	Chao1	1055.43±95.03b	1305.34±14.97a	1242.85±39.17a	1326.90±14.39a
Fungus	ACE	185.41±40.11b	279.63±13.94a	281.73±29.52a	263.26±9.65a
	Chao1	176.25±35.13a	251.77±13.57a	256.58±37.65a	262.41±11.21a

^a^ Data represent mean ± standard error of 3 replicates. Different letters on the same line indicate significant differences among treatments as determined by the Duncan test (P<0.05).CK, control; ASB, aboveground soybean parts; USB, underground soybean parts; WSB, whole soybean plants.

**Fig 3:**
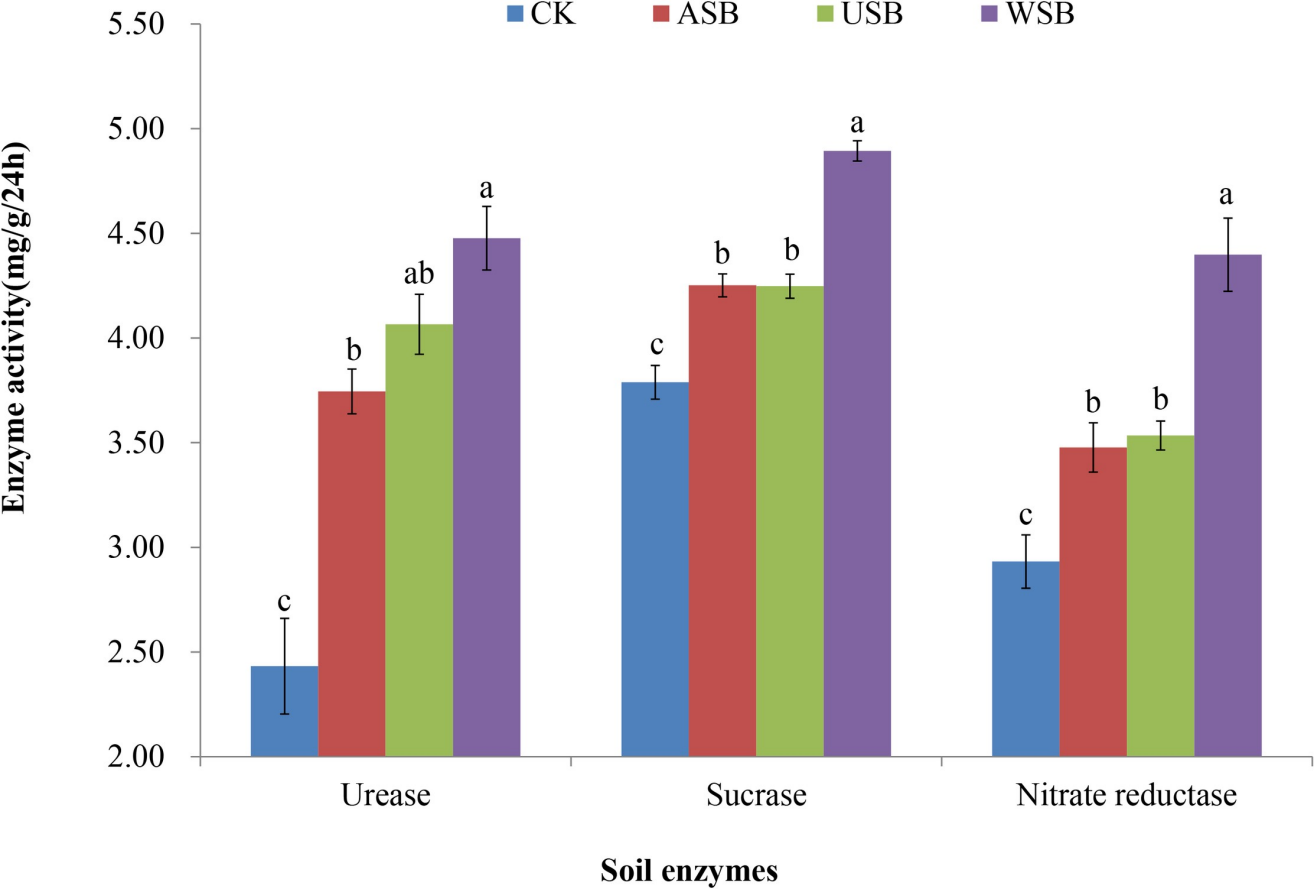
Activities of enzymes in soils treated with consecutive soybean planting and covering. Activities of the enzymes were measure at the conclusion of the third application of the treatments. Bars represent mean ± standard error. Different lowercase letters indicate significant differences among treatments (P<0.05). CK, control; ASB, aboveground soybean parts; USB, underground soybean parts; WSB, whole soybean plants.

Activities of three important microbial enzymes in soil, urease, sucrase, and nitrate reductase, were measured after the completion of the third application of the treatments (Fig 3, S8 Table in S1 File). The activities of these enzymes were a measure of the soil microbial metabolism, and thus they served as a good indicator of the microbial abundance in the soil. In comparison to the control, activities of these three enzymes increased significantly in all the treatments, indicating that both the soybean roots and aboveground parts stimulated the proliferation of soil microbiome, as indicated by the increase in the enzymatic activities. All the enzymes were most active in the WSB treatment, with the activities of the sucrase and nitrate reductase significantly higher than in the ASB and USB treatments. It was interesting to note that there were significantly higher soil nitrate reductase activities in the WSB treatment (4.40±0.18 mg/g/24h) than those in the USB treatment (3.53±0.07mg/g/24h), although though both treatments contained the soybean underground root residues in the soil.

The increase in soil microbiome abundance was also corroborated by the significant increase in the content of soil organic matter (SOM) (S4 and S9 Tables in S1 File). The dry soybean matter for the ASB and WSB treatments weighted respectively at 68.59±5.83g and 75.60±6.34g per pot, much more than that of USB treatment at 3.85±0.56g per pot. After the completion of the third application of all the treatments, the contents of SOM were measured at 1.30±0.09%, 1.76±0.04%, 1.67±0.03%, and 2.03±0.04%, respectively in the CK, ASB, USB, and WSB treatments. ANOVA and Duncan multiple range test showed that all soybean planting and covering treatments had significantly increased SOM (S9 Table in S1 File), with the SOM contents significantly higher (P<0.05) in the three treatments than in CK, and significantly higher in the WSB treatment than in the ASB and USB treatments. The contents of SOM were positively correlated with the amounts of dry soybean matters, soil microbial richness (Table 3), and soil enzymatic activities (Fig 3). The small difference in the content of SOM, despite the large difference in the amount of soybean dry matters between the ASB and USB treatment, suggested that soybean root systems and root residues were highly active in promoting soil microbial growth, leading to a higher rate of SOM conversion.

### Correlations of exchangeable cations and organic matter to the soil pH

**Fig 4:**
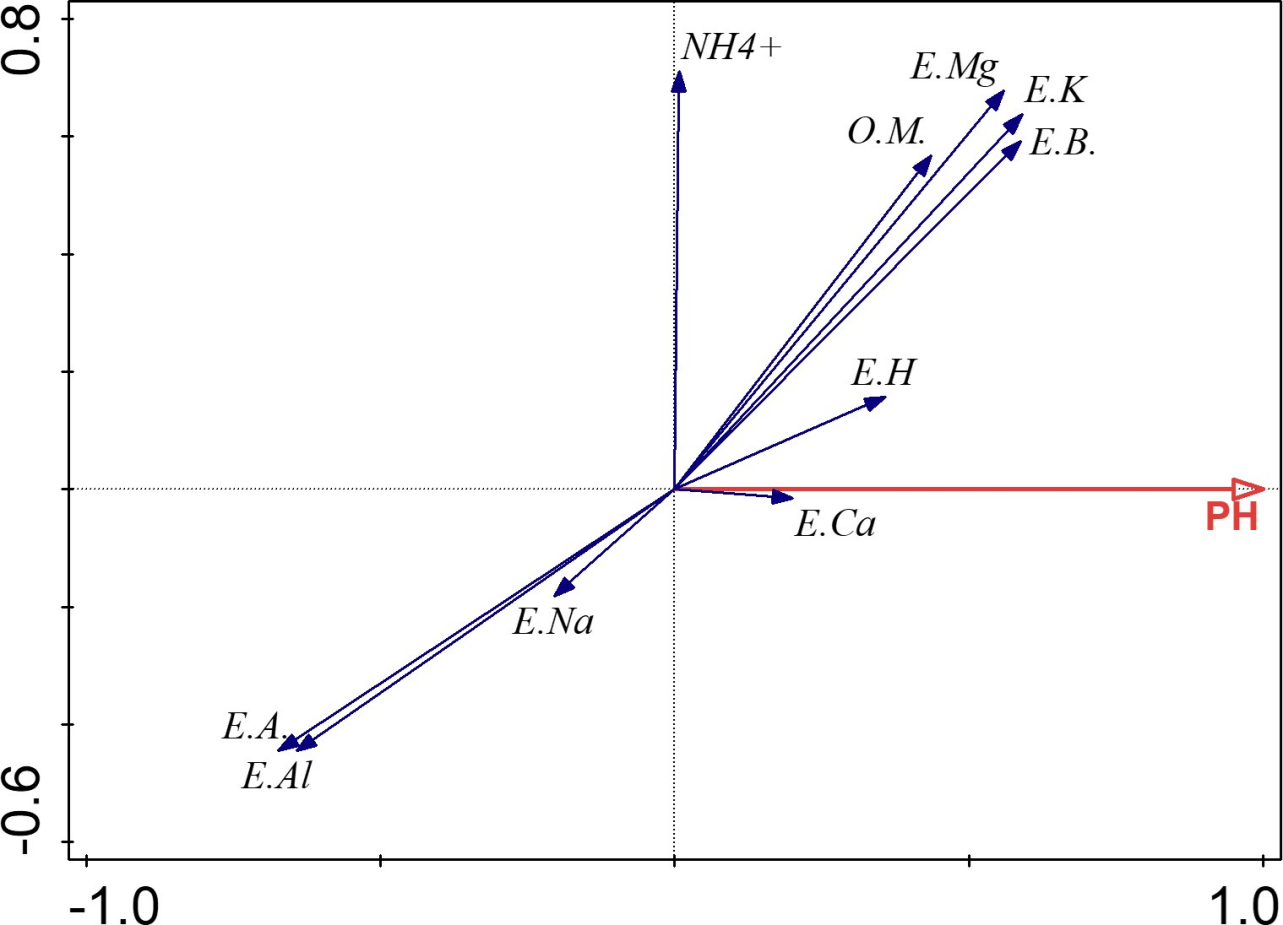
Correlations of exchangeable cations and organic matter to the soil pH. Various soil characteristics are used as independent variables to assess their contribution to the dependent variable, soil pH, in this redundancy analysis. E.Al = exchangeable Al^3+^, E.H = exchangeable H^+^, E.A = exchangeable acidity (E.Al + E.H), E.K = exchangeable K^+^, E.Na = exchangeable Na^+^, E.Ca = exchangeable Ca^2+^, E.Mg = exchangeable Mg^2+^, E.B = exchangeable bases (E.K + E.Na + E.Ca + E.Mg), O.M = organic matter of soil.

Redundancy analysis [27] was conducted using the dataset collected at the last time point (12/31/2018) to identify key factors that influenced the soil pH, with pH values as a dependent variable and soil characteristics as independent variables (Fig 4). The exchangeable base, exchangeable Mg^2+^ and K^+^, and organic matter are significantly and positively correlated to the soil pH; while the exchangeable total acidity and exchangeable Al^3+^ are significantly and negatively correlated. Ca^2+^ and H^+^ played a positive but unsubstantial role in increasing the soil pH. These data showed that the tea garden soil pH was determined largely by exchangeable Mg^2+^, K^+^, Al^3+^ and organic matter.

## Discussion

In this study, we demonstrated differential effects of the aboveground parts and the underground root system of soybeans on the soil pH, effectively addressing the controversy of planting legume crops on increasing soil acidification in theory but improving soil pH in practice. Treatments with the aboveground soybean (ASB) parts and the whole soybean (WSB) plants significantly increased the soil pH while the treatment with the underground soybean (USB) root system had negative but insignificant effect on the soil pH (Fig 1). These effects were mediated by the changes in the increase of specific exchangeable bases, particularly exchangeable K^+^ & Mg^2+^, and in the decrease of specific exchangeable acidity, specifically exchangeable Al^3+^. The increase of the soil organic matter (SOM) contents brought about by increased microbial richness and abundance also had a significant impact on the soil pH.

A major finding of this study is the differential roles of the aboveground and underground soybean parts played in effecting the soil pH (Fig 1). The contribution of aboveground soybean parts to the increase in the soil pH is well supported from previous studies. Soil covering with the aboveground residues of canola, chickpea and wheat has been reported to increase the soil pH due to the sequestering of H^+^ by the added organic matters and alkalinity released from the ammonification and decarboxylation processes during the degradation of the organic matter [29]. Similar results have also been reported with faba bean and wheat residues as a soil cover [30].

The contribution of the underground soybean root system to a decrease in the soil pH is a somewhat novel finding from this study. The nitrification of NH_4_^+^ fixed by root-associated rhizobial species could be a major contributor to the decreased soil pH. Nitrification of NH_4_^+^ into NO_3_^-^ have been found to increase the H^+^ concentration in the soil [8, 9]. This is consistent with the finding of less NH_4_^+^ and more NO_3_^-^ in the USB treatment than in the WSB treatment in this study (Fig 2). The increase in NH_4_^+^ in the WSB treatment could be explained by the significantly higher activities of soil nitrate reductase in the WSB treatment than in the USB treatment (Fig 3). Nitrate reductase catalyzes the conversion of NO_3_^-^ to NO_2_^-^, which is then converted to NH_4_^+^ (NH_3_) [31]. Similar increases in NH_4_^+^ were also observed in soybean–tea intercropping [32], and in soils with lupin and wheat plant materials incorporated [33].

NH_4_^+^ in the USB treatment was relatively unchanged in comparison with the CK treatment even though the amount of NH_4_^+^ fixed by the rhizobial nodules should be expected at a similar level as that in the WSB treatment. It is possible that a substantial portion of fixed NH_4_^+^ has been transferred to the aboveground parts of soybeans or transformed into NO_3_^-^, a process that also releases H^+^ to reduce the soil pH of USB [8, 9].

Decomposition of the larger amount of soybean dry matters could possibly explain the observed changes in the exchangeable acidity and bases in the ASB and WSB treatment (Table 1). Humus degraded from soybean plants adds the amorphous specific surface area of soil colloids and raises the soil cation exchange capacity, which increase exchangeable base cations and decrease exchangeable acidic cations [34]. A similar mechanism was proposed for the increased pH in soils covered with legume, wheat, and rice plant materials [35, 36]. The increase in exchangeable base and the decrease in exchangeable acid can increase soil pH, therefore improving soil acidification [37].

Measurements of exchangeable cations identified Mg^2+^ and K^+^ as major players in the increase of the exchangeable bases and the exchangeable Al^3+^ as a major contributor in the decrease of the exchangeable acidity (Table 2). The exchangeable acidity and the exchangeable Al^3+^ but not the exchangeable H^+^ were found to be significantly and negatively correlated to the soil pH in this study (Fig 4), consistent with a previously report [38]. The exchangeable base, exchangeable Mg^2+^ and K^+^, and organic matter are significantly and positively correlated to the soil pH. This agrees with previous reports that the main buffering substances for acidity in the soil between pH 4.2 and 5.0 included exchangeable base cations [39] and were affected by organic matters [40]. The dramatic increase in exchangeable K^+^ and Mg^2+^ could be attributed to soybean planting and covering. Soybean straw were reported to contain abundant K^+^ and Mg^2+^ at 16.21 cmol/kg and 17.86 cmol/kg, respectively [38], which could be converted to exchangeable K^+^ and Mg^2+^ in soil after decomposition of soybean residues. Soybean planting was previously shown to improve soil physicochemical properties and increase the exchangeable K^+^ and Mg^2+^ [11, 32].

All the soybean planting and covering treatments significantly increased the abundance of soil bacteria and fungi, activities of enzymes in soil, and SOM contents in this study. SOM has been reported to sequester Al^3+^, which reduces the concentration of exchangeable Al^3+^ and improves the acidity of soil [41]. The increased richness and metabolic activities of microbes in the soil can promote degradation of plant residues to form humus and improved the colloid structure of the soil, which further enhances the ability of soil to absorb H^+^ and released OH^-^ in the soil solution [13], and eventually increase the soil pH [12]. Similar mechanisms have been proposed to account for the interactive effects on the soil pH, types of plant residues, and microbes [42] and for the effect of organic fertilizers on soil microbial communities [28].

The whole soybean plants contained more dry matters and therefore increased the soil SOM the most, in agreement with the results of Duan et al. [32]. Soybean plant residues were also reported to serve as an organic fertilizer to increase the soil pH [43]. Despite the fact that soybean root residues contained a very small amount of dry matters, the SOM in the USB treatment still increased significantly to a high level. This demonstrated the importance of a very active microbiome and microbial metabolic activities in the rhizosphere promoted by soybean roots. The higher SOM content in the USB treatment than in CK, however, had no effect on the soil pH. One plausible explanation is that the beneficial effect of SOM had been offset by the increased H^+^ produced from NH_4_^+^ fixed by the rhizobial community in the root system of soybeans.

It was interesting to note that the soil pH in the controls also increased by 0.28 unit, from 4.36±0.01 at the beginning of the study to 4.64±0.02 at the end of the study. This was likely caused by the use of the basic fertilizer (pH 9.7) and irrigation with weak basic water (pH 7.4) during the experiments. Another limitation of this study is that these effects were evaluated only in tea garden soil in pots. This approach was designed to avoid the complications caused by large variations in soil properties within a tea garden and between tea gardens. Further studies are needed to dissect further the differential effects of the aboveground and the underground parts of soybeans on the soil pH in tea gardens.

## Conclusions

Soybean planting increased the pH of the acidified tea garden soil. The aboveground parts of soybean plant contributed the most to the increase of the soil pH while the soybean roots played little if any role in effecting the soil pH. The change in the pH of the tea garden soil was mediated through the increase in the exchangeable bases and the decrease in the exchangeable acidity. Exchangeable Mg^2+^ and K^+^ played key roles in the changes in the total exchangeable bases while the exchangeable Al^3+^ in the exchangeable acidity. The increased richness and abundance in soil microbiome promoted by soybean planting led to higher metabolic activities and consequently higher soil organic matters, resulting in the improvement of the soil acidity.

## Supporting information

S1 File Tables of original data and statistical analysis used in this study. (PDF)

## References

[1] Ruan JY, Gerendás J, Härdter R, Sattelmacher B. Effect of nitrogen form and root-zone pH on growth and nitrogen uptake of tea (*Camellia sinensis*) plants. Annals of Botany. 2007; 99: 301–10. doi: 10.1093/aob/mcl258 17204540 PMC2802997

[2] Yan P, Wu LQ, Wang DH, Fu JY, Chen S, Li X, et al. Soil acidification in Chinese tea plantations. Science of the Total Environment. 2020;715:136963–7. doi: 10.1016/j.scitotenv.2020.136963 32014781

[3] Wang X, Kato T, Tokuda S. Environmental problems caused by heavy application of nitrogen fertilisers in Japanese tea fields. In: Land Degradation. Berlin, Germany: Springer Netherlands; 2001. p.141–50.

[4] Alekseeva T, Alekseev A, Xu RK, Zhao AZ, Kalinin P. Effect of soil acidification induced by a tea plantation on chemical and mineralogical properties of Alfisols in eastern China. Environ Geochem Health. 2011;33:137–48. doi: 10.1007/s10653-010-9327-5 20563880

[5] Zhang MK, Zhou C, Huang CY. Relationship between extractable metals in acid soils and metals taken up by tea plants. Communications in Soil Science and Plant Analysis. 2006;37:347–61.

[6] Liu LT, Knight DJ, Lemke RL, Richard E, Farrell RE. A side-by-side comparison of biological nitrogen fixation and yield of four legume crops. Plant Soil. 2019;442:169–82.

[7] Procházka P, Štranc P, Vostřel J, Řehoř J, Křováček J, Brinar J, et al. The influence of effective soybean seed treatment on root biomass formation and seed production. Plant, Soil and Environment. 2019;65:588–93.

[8] Cregan PD, ScottB J. Soil acidification-an agricultural and environmental problem. In: Pratley JE, Robertson A ed. Agriculture and the Environmental Imperative. Melbourne, Australia: CSIRO Publishing; 1998. p.98–128.

[9] Tang C, Unkovich MJ, Bowden JW. Factors affecting soil acidification under legumes. III. Acid production by N_2_ –fixing legumes as influenced by nitrate supply. New Phytol. 1999;143:513–21. doi: 10.1046/j.1469-8137.1999.00475.x 33862886

[10] Ruan JY, Ma LF, Shi YZ. Aluminium in tea plantations: mobility in soils and plants, and the influence of nitrogen fertilization. Environmental Geochemistry and Health. 2006;28(6):519–28. doi: 10.1007/s10653-006-9047-z 16826449

[11] Li JL, Tu PN, Chen N, Tang JC, Wang XR, Nian H, et al. Effects of Tea Intercropping with Soybean. China Agricultural Science. 2008:(7):2040–7. Chinese.

[12] Yang HB, Li ZL, Xu Z, Deng M, Sheng ZL. Effects of Intercropping Green Manure on Soil Available Zinc and Nutrient Content of Young Tea Garden. Chinese Agricultural Science Bulletin. 2018;34(17):99–103. Chinese.

[13] Luo YP. Tea cultivation.2th ed. Beijing, China: China Agricultural Press; 2013. Chinese.

[14] Bao SD. Analysis of soil agrochemical, 3rd ed. Beijing, China: China Agricultural Press; 2000. Chinese.

[15] Castrillo G, Teixeira PJPL, Paredes SH, Law TF, Lorenzo LD, Feltcher ME, et al. Root microbiota drive direct integration of phosphate stress and immunity. Nature. 2017;543:513–18. doi: 10.1038/nature21417 28297714 PMC5364063

[16] Magoc T, Salzberg S. FLASH: Fast length adjustment of short reads to improve genome assemblies. Bioinformatics. 2011; 27(21): 2957–63. doi: 10.1093/bioinformatics/btr507 21903629 PMC3198573

[17] Bolger AM, Lohse M, Usadel B. Trimmomatic: a flexible trimmer for Illumina sequence data. Bioinformatics. 2014; 30(15): 2114–20. doi: 10.1093/bioinformatics/btu170 24695404 PMC4103590

[18] Edgar RC, Haas BJ, Clemente JC, Quince C, Knight R. UCHIME improves sensitivity and speed of chimera detection. Bioinformatics. 2011; 27(16):2194–200. doi: 10.1093/bioinformatics/btr381 21700674 PMC3150044

[19] Edgar Robert C. UPARSE: highly accurate OTU sequences from microbial amplicon reads. Nature Methods. 2013; 10(10): 996–8. doi: 10.1038/nmeth.2604 23955772

[20] Quast C, Pruesse E, Yilmaz P, Gerken J, Schweer T, Yarza P, et al. The SILVA ribosomal RNA gene database project: improved data processing and web-based tools. Nucleic Acids Research, 2012. doi: 10.1093/nar/gks1219 23193283 PMC3531112

[21] Kõljalg U, Nilsson RH, Abarenkov K, Tedersoo L, Taylor AF, Bahram M, et al.Towards a unified paradigm for sequence‐based identification of fungi. Molecular Ecology. 2013;22(21):5271–7. doi: 10.1111/mec.12481 24112409

[22] Liu LY, Li CZ, Zhu SH, Xu Y, Li HY, Zheng XQ, et al. Combined application of organic and inorganic nitrogen fertilizers affects soil prokaryotic communities compositions. Agronomy. 2020;10:132.

[23] Ma YH, Fu SL, Zhang XP, Zhao K, Chen HYH. Intercropping improves soil nutrient availability, soil enzyme activity and tea quantity and quality. Applied Soil Ecology. 2017; 119:171–8.

[24] Liu X, Wang J, Zhao X. Effects of simulated nitrogen deposition on the soil enzyme activities in a *Pinus tabulaeformis* forest at the Taiyue Mountain. Acta Ecologica Sinica. 2015;35:4613–24.

[25] Peng CJ, Li Q, Gu HH, Song ZX. Effects of simulated nitrogen deposition and management type on soil enzyme activities in Moso bamboo forest. Chinese Journal of Applied Ecology. 2017;28:423–9.Chinese. doi: 10.13287/j.1001-9332.201702.001 29749149

[26] Schloss PD, Westcott SL, Ryabin T, Hall JR, Hartmann M, Hollister EB, et al. Introducing mothur: open-source, platform-independent, community-supported software for describing and comparing microbial communities. Applied and Environmental Microbiology. 2009;75(23):7537–41. doi: 10.1128/AEM.01541-09 19801464 PMC2786419

[27] Braak ter CJF, Smilauer P. Canoco reference manual and user’s guide: software of ordination (version 5.0). Ithaca, NY, USA: Microcomputer Power; 2012.

[28] Bolan NS, Hedley MJ, White RE. Processes of soil acidification during nitrogen cycling with emphasis on legume based pastures. Plant and Soil. 1991;134:53–63.

[29] Butterly C, Baldock J, Tang C. The contribution of crop residues to changes in soil pH under field conditions. Plant and Soil. 2013;366:185–98.

[30] Yan F, Schubert S. Soil pH changes after application of plant shoot materials of faba bean and wheat. Plant and Soil. 2000;220:279–87.

[31] Barro F, Fontes AG, Maldonado JM. Organic nitrogen content and nitrate and nitrite reductase activities in tritordeum and wheat grown under nitrate or ammonium. Plant and Soil. 1991;135:251–6.

[32] Duan Y, Shen JZ, Zhang XL, Bo W, Ma YC, Wang Y, et al. Effects of soybean-tea intercropping on soil-available nutrients and tea quality. Acta Physiologiae Plantarum. 2019;41:140–9.

[33] Xu RK, Coventry DR. Soil pH changes associated with lupin and wheat plant materials incorporated in a red–brown earth soil. Plant and Soil. 2003;250:113–9.

[34] Sumner ME. Handbook of soil science. Boca Raton, London, New York, Washington, D.C., USA: CRC Press LLC; 2000.

[35] Tang C, Yu Q. Impact of chemical composition of legume residues and initial soil pH on pH change of a soil after residue incorporation. Plant and Soil. 1999;215:29–38.

[36] Wang L, Wang Y, Yang XL, Zhang M, Jiang X. Use of Crop Residues to Ameliorate Soil Acidity in A Tea Garden Soil. Soils. 2013;45(3):430–6. Chinese.

[37] Wang H, Xu RK, Wang N, Li XH. Soil Acidification of Alfisols as Influenced by Tea Cultivation in Eastern China. Pedosphere. 2010; 20(6):799–806.

[38] Wang N, Li JY, Xu RK. Use of agricultural by-products to study the pH effects in an acid tea garden soil. Soil Use and Management. 2009;25:128–32.

[39] Ulrich B. Natural and anthropogenic components of soil acidification. Z. Pflanzenernähr. Bodenk. 1986;149:717–20.

[40] Balík J, Kulhánek M, Černý J, Sedlář O, Suran P. Impact of organic and mineral fertilising on aluminium mobility and extractability in two temperate Cambisols. Plant, Soil and Environment. 2019;65:581–7.

[41] Naramabuye F, Haynes R. Short-term effects of three animal manures on soil pH and Al solubility. Soil Research. 2006;44(5):515–21.

[42] Zhang KL, Chen L, Li YF, Brookes PC, Xu JM, Luo Y. Interactive effects of soil pH and substrate quality on microbial utilization. European Journal of Soil Biology. 2020;96:103151–9.

[43] Lin W, Lin M, Zhou H, Wu H, Li Z, Lin W. The effects of chemical and organic fertilizer usage on rhizosphere soil in tea orchards. PLoS ONE. 2019;14(5):e0217018. doi: 10.1371/journal.pone.0217018 31136614 PMC6538140

