# Supplementary material for: Consecutive soybean (Glycine max) planting and covering improve acidified tea garden soil
Source: PLoS One. 2021 Jul 13;16(7):e0254502. doi: 10.1371/journal.pone.0254502 (PMC8277052; doi:10.1371/journal.pone.0254502)
Supplement: S1 File — (PDF) [file pone.0254502.s001.pdf]

S1 Table. Original data of soil pH and soil exchangeable cations sampled on 09/09/2017 (cmol/kg except pH)*

Treat-ment**	pH	Exchange-able Al^{3+}	Exchange-able H^+	Exchange-able acid	Exchange-able K^+	Exchange-able Na^+	Exchange-able Ca^{2+}	Exchange-able Mg^{2+}	Exchange-able base
CK	4.53	2.319	0.361	2.680	1.101	1.507		0.207	
CK	4.56	2.371	0.361	2.732	1.052	1.301	8.053	0.264	10.670
CK	4.56	1.753	0.412	2.165	0.755	1.595	8.033	0.477	10.860
CK	4.57	2.010	0.412	2.422	0.903	1.801	7.986	0.507	11.197
CK	4.47	2.423	0.412	2.835	0.722	1.301	8.132	0.184	10.339
CK	4.49	2.371	0.464	2.835	0.771	1.213	8.055	0.134	10.173
CK	4.50	2.577	0.412	2.990	1.118	1.154	8.030	0.336	10.638
CK	4.54	2.371	0.412	2.783	0.788	1.448	8.085	0.396	10.717
ASB	4.56	2.216	0.515	2.732	1.052	0.683	8.072	0.381	10.188
ASB	4.53	2.165	0.464	2.629	1.151	0.683	8.128	0.354	10.316
ASB	4.60	2.371	0.618	2.989	0.738	0.654	8.087	0.297	9.776
ASB	4.55	2.526	0.619	3.144	0.787	0.771	8.103	0.442	10.104
ASB	4.52	2.216	0.309	2.526	1.200	0.742	8.144	0.524	10.610
ASB	4.58	2.165	0.464	2.629	0.721	1.007	8.152	0.518	10.398
ASB	4.42	2.268	0.464	2.732	0.754	0.654	8.083	0.463	9.955
ASB	4.45	2.216	0.464	2.680	0.490	0.654	8.139	0.402	9.685
ASB	4.53	2.268	0.515	2.783	1.085	1.507	8.023	0.391	11.007
ASB	4.54	2.320	0.515	2.835	1.184	1.507	8.029	0.336	11.056
ASB	4.54	2.216	0.412	2.629	0.837	1.478	8.017	0.546	10.878
ASB	4.56	2.113	0.412	2.526	0.771	1.301	8.055	0.587	10.715
ASB	4.49	2.216	0.464	2.680	0.870	1.448	8.015	0.436	10.768
ASB	4.47	2.216	0.412	2.629	0.854	1.595	8.239	0.490	11.178
ASB	4.65	1.959	0.412	2.371	0.887	1.419	8.018	0.557	10.881
ASB	4.68	1.958	0.361	2.319	0.837	1.390	8.056	0.546	10.829
USB	4.62	1.907	0.309	2.216	0.474	0.683		0.662	
USB	4.63	2.268	0.309	2.577	0.821	0.889	8.214	0.562	10.486
USB	4.53	2.371	0.567	2.938	0.738	0.742	8.072	0.708	10.260
USB	4.52	2.319	0.515	2.835	0.391	0.712	8.118	0.604	9.825
USB	4.56	2.629	0.412	3.041	0.523	0.657	8.129	0.271	9.579
USB	4.55	2.423	0.412	2.835	0.325	0.654	8.110	0.274	9.363
USB	4.55	2.319	0.412	2.732	0.441	0.713	8.106	0.337	9.597
USB	4.57	2.423	0.361	2.784	0.606	0.565	7.929	0.352	9.452
USB	4.51	1.752	0.258	2.010	0.523	1.242	7.985	0.469	10.219
USB	4.53	1.701	0.309	2.010	1.101	1.360	8.032	0.529	11.022
USB	4.55	2.216	0.618	2.835	0.788	1.272	8.130	0.380	10.570

USB	4.54	2.165	0.515	2.680	0.804	1.272	8.085	0.403	10.565
USB	4.50	2.268	0.412	2.680	0.672	1.301	8.060	0.299	10.332
USB	4.51	2.474	0.361	2.835	0.689	1.360	8.057	0.330	10.436
USB	4.56	1.907	0.412	2.319	0.689	1.389	8.075	0.413	10.566
USB	4.57	2.165	0.309	2.474	0.639	1.242	8.057	0.455	10.394
WSB	4.50	2.680	0.412	3.092	0.523	0.830	8.127	0.286	9.767
WSB	4.52	2.423	0.412	2.835	0.425	0.624	8.069	0.264	9.381
WSB	4.56	2.526	0.515	3.041	0.523	0.595	8.013	0.586	9.717
WSB	4.49	2.474	0.464	2.938	0.606	0.801	8.134	0.658	10.199
WSB	4.46	2.680	0.464	3.144	0.854	0.859	8.130	0.563	10.406
WSB	4.49	2.680	0.515	3.196	0.804	0.683		0.465	
WSB	4.70	1.855	0.258	2.113	0.622	0.712	8.126	0.546	10.006
WSB	4.66	1.907	0.258	2.165	0.689	0.742	8.154	0.564	10.149
WSB	4.47	2.474	0.619	3.093	0.969	1.595	8.049	0.579	11.193
WSB	4.47	2.474	0.567	3.041	0.986	1.625	8.097	0.545	11.254
WSB	4.53	2.216	0.412	2.629	0.986	1.507	8.203	0.253	10.948
WSB	4.55	1.959	0.464	2.423	0.804	1.448	8.101	0.270	10.623
WSB	4.45	1.649	0.309	1.959	0.738	1.507	8.034	0.346	10.625
WSB	4.48	1.804	0.258	2.062	0.754	1.536	8.081	0.340	10.711
WSB	4.57	2.062	0.258	2.319	0.804	1.537	8.160	0.402	10.903
WSB	4.55	2.010	0.361	2.371	0.672	1.419	8.110	0.387	10.587

* Exchangeable acidity = Exchangeable Al^{3+} + Exchangeable H^{+}; Exchangeable bases = Exchangeable K^{+} + Exchangeable Na^{+} + Exchangeable Ca^{2+} + Exchangeable Mg^{2+}.

** CK, control; ASB, aboveground soybean parts; USB, underground soybean parts; WSB, whole soybean plants.

S2 Table. Original data of soil pH and soil exchangeable cations sampled on 05/07/2018 (cmol/kg except pH)*

Treat-ment**	pH	Exchange-able Al^{3+}	Exchange-able H^{+}	Exchange-able acid	Exchange-able K^{+}	Exchange-able Na^{+}	Exchange-able Ca^{2+}	Exchange-able Mg^{2+}	Exchange -able base
CK	4.53	2.301	0.409	2.710	0.912	1.115	8.044	0.243	10.314
CK	4.52	2.301	0.409	2.710	0.880	1.028	8.148	0.250	10.306
CK	4.63	1.582	0.459	2.041	0.911	0.968	8.131	0.487	10.496
CK	4.65	1.582	0.408	1.990	1.025	1.375	8.238	0.528	11.166
CK	4.55	2.494	0.204	2.698	0.990	1.052	7.870	0.193	10.106
CK	4.53	2.444	0.255	2.698	0.892	1.111	8.161	0.222	10.386
CK	4.63	2.596	0.204	2.800	0.827	0.995	8.137	0.284	10.243
CK	4.61	2.495	0.255	2.749	0.957	1.111	8.211	0.346	10.625
ASB	4.62	1.472	0.508	1.980	1.687	0.644	8.042	0.502	10.875
ASB	4.61	1.625	0.457	2.082	2.109	0.760	8.014	0.499	11.382
ASB	4.71	1.672	0.355	2.027	1.147	0.671	7.940	0.304	10.063
ASB	4.76	1.723	0.355	2.077	1.099	0.527	8.000	0.422	10.048
ASB	4.80	1.220	0.203	1.424	2.096	0.790	7.781	0.711	11.378
ASB	4.77	1.169	0.203	1.373	1.151	0.674	7.937	0.791	10.553
ASB	4.65	1.678	0.509	2.187	1.608	0.645	8.012	0.566	10.830
ASB	4.62	1.678	0.458	2.136	0.744	0.645	7.851	0.288	9.529
ASB	4.56	2.250	0.205	2.455	1.044	1.145	8.462	0.447	11.098
ASB	4.58	2.250	0.256	2.506	1.224	1.203	8.476	0.450	11.353
ASB	4.70	1.471	0.152	1.623	2.303	1.107	8.669		
ASB	4.72	1.471	0.203	1.674	2.351	1.107	8.466		
ASB	4.55	1.930	0.457	2.387	1.671	1.050	8.347	0.498	11.566
ASB	4.60	1.879	0.457	2.336	1.557	1.079	8.459	0.579	11.673
ASB	4.62	1.673	0.456	2.129	1.538	1.135	8.645	0.671	11.988
ASB	4.57	1.672	0.507	2.179	1.472	1.076	8.536	0.669	11.753
USB	4.64	1.634	0.408	2.042	1.222	1.580	7.985	0.342	11.129
USB	4.65	1.532	0.460	1.991	1.320	1.406	7.996	0.357	11.079
USB	4.66	2.031	0.406	2.437	0.353	0.673	7.555	0.337	8.917
USB	4.68	2.031	0.355	2.386	0.450	0.702	8.232	0.360	9.744
USB	4.66	2.090	0.357	2.447	0.926	1.258	7.559	0.288	10.032
USB	4.65	2.142	0.357	2.499	0.893	1.083	7.935	0.309	10.221
USB	4.68	1.533	0.460	1.993	1.206	1.406	7.806	0.428	10.847
USB	4.66	1.533	0.409	1.941	1.370	1.319	8.196	0.473	11.357
USB	4.69	1.998	0.359	2.356	0.684	0.913	8.478	0.405	10.481
USB	4.77	1.998	0.410	2.408	0.750	0.971	8.526	0.401	10.648
USB	4.65	2.295	0.459	2.754	0.583	0.821	8.651	0.496	10.552

USB	4.54	2.397	0.408	2.805	0.779	0.967	8.638	0.553	10.937
USB	4.58	2.085	0.305	2.390	0.565	0.790	8.516	0.490	10.362
USB	4.59	1.932	0.407	2.339	0.679	0.819	8.504	0.514	10.516
USB	4.53	2.145	0.306	2.451	0.748	1.522	8.392	0.280	10.941
USB	4.56	2.145	0.255	2.400	0.895	1.493	8.422	0.337	11.147
WSB	4.64	2.411	0.410	2.821	1.573	1.002	8.628	0.355	11.558
WSB	4.56	2.360	0.359	2.719	1.638	1.676	8.350	0.277	11.942
WSB	4.54	1.578	0.356	1.935	1.822	0.994	8.467	0.464	11.747
WSB	4.53	1.629	0.407	2.037	1.691	0.907	8.808	0.582	11.988
WSB	4.60	1.832	0.356	2.188	1.446	0.936	8.452	0.512	11.346
WSB	4.63	1.832	0.356	2.188	1.511	1.052	8.465	0.531	11.558
WSB	4.75	1.687	0.358	2.044	1.583	1.203	8.693	0.673	12.151
WSB	4.73	1.635	0.409	2.044	1.436	1.144	8.716		
WSB	4.71	1.677	0.356	2.033	1.492	0.760	7.887	0.421	10.560
WSB	4.73	1.626	0.407	2.033	1.655	0.760	8.068	0.501	10.985
WSB	4.66	1.743	0.410	2.153	1.703	0.709	8.045	0.416	10.873
WSB	4.68	2.102	0.359	2.461	1.736	0.767	8.121	0.406	11.030
WSB	4.56	2.232	0.457	2.689	1.799	0.759	7.968	0.463	10.989
WSB	4.54	2.181	0.507	2.689	2.221	0.759	8.124	0.518	11.623
WSB	4.61	2.238	0.458	2.696	2.536	0.993	8.052	0.598	12.179
WSB	4.63	2.187	0.509	2.696	1.853	0.906	8.037	0.570	11.366

* Exchangeable acidity = Exchangeable Al^{3+} + Exchangeable H^{+}; Exchangeable bases = Exchangeable K^{+} + Exchangeable Na^{+} + Exchangeable Ca^{2+} + Exchangeable Mg^{2+}.

** CK, control; ASB, aboveground soybean parts; USB, underground soybean parts; WSB, whole soybean plants.

S3 Table. Original data of soil pH and soil exchangeable cations sampled on 12/31/2018 (cmol/kg except pH)*

Treat-ment**	pH	Exchange-able Al^{3+}	Exchange-able H^+	Exchange-able acid	Exchange-able K^+	Exchange-able Na^+	Exchange-able Ca^{2+}	Exchange-able Mg^{2+}	Exchange -able base
CK	4.68	2.225	0.414	2.639	0.360	1.217	8.458	0.101	10.136
CK	4.69	2.380	0.207	2.587	0.393	1.158	8.464	0.118	10.134
CK	4.61	2.268	0.206	2.475	0.573	1.566	8.090	0.276	10.506
CK	4.62	2.320	0.258	2.578	0.491	1.596	8.024	0.271	10.382
CK	4.58	2.477	0.258	2.736	0.491	1.008	8.392	0.091	9.982
CK	4.59	2.375	0.258	2.633	0.425	1.303	8.465	0.117	10.310
CK	4.70	2.323	0.310	2.632	0.673	1.716	8.275	0.314	10.978
CK	4.63	2.374	0.310	2.684	0.541	1.480	8.258	0.332	10.611
ASB	4.72	2.114	0.309	2.423	1.564	1.331	8.370	0.752	12.017
ASB	4.83	1.805	0.309	2.114	1.399	1.213	8.409	0.779	11.800
ASB	4.64	2.376	0.258	2.634	1.137	1.215	8.121	0.465	10.938
ASB	4.61	1.912	0.258	2.170	1.219	1.156	8.202	0.485	11.063
ASB	4.70	2.119	0.362	2.481	1.071	1.216	7.957	0.544	10.787
ASB	4.66	1.964	0.258	2.222	1.021	1.157	8.242	0.564	10.984
ASB	4.74	1.856	0.258	2.114	1.580	1.301	8.392	0.171	11.444
ASB	4.75	1.959	0.309	2.268	1.614	1.272	8.339	0.189	11.414
ASB	4.71	2.325	0.207	2.532	1.104	1.363	8.782	0.503	11.752
ASB	4.75	2.377	0.258	2.635	1.187	1.215	8.760	0.568	11.731
ASB	4.75	2.372	0.258	2.630	1.383	1.096	8.548	0.548	11.574
ASB	4.74	2.166	0.258	2.424	1.548	1.096	8.729	0.582	11.954
ASB	4.81	1.910	0.258	2.168	1.169	1.067	8.339	0.776	11.350
ASB	4.82	2.064	0.103	2.168	1.086	1.067	8.399	0.773	11.325
ASB	4.65	2.583	0.258	2.841	1.435	1.599	8.630	0.731	12.395
ASB	4.71	2.273	0.258	2.531	1.451	1.510	9.063	0.740	12.765
USB	4.62	2.941	0.155	3.096	0.722	1.185	8.590	0.256	10.753
USB	4.62	2.425	0.103	2.528	0.590	1.096	8.685	0.298	10.669
USB	4.61	2.377	0.207	2.584	1.005	1.599	8.518	0.269	11.391
USB	4.59	2.171	0.258	2.429	1.137	1.599	8.639	0.332	11.708
USB	4.64	2.333	0.259	2.592	0.776		8.571	0.457	
USB	4.57	2.177	0.207	2.385	0.726	1.161	8.518	0.423	10.828
USB	4.60	2.374	0.206	2.580	0.788	1.037	8.486	0.395	10.706
USB	4.64	2.425	0.103	2.529	0.557	1.037	8.187	0.382	10.165
USB	4.61	2.529	0.155	2.684	0.607	1.332	8.249	0.294	10.482
USB	4.54	2.477	0.155	2.632	0.524	1.361	8.264	0.325	10.474
USB	4.57	2.376	0.155	2.530	0.706	1.097	8.230	0.398	10.432

USB	4.56	2.376	0.207	2.582	0.839	1.127	8.673	0.427	11.065
USB	4.65	2.222	0.155	2.377	0.475	1.393	8.331	0.467	10.666
USB	4.65	2.377	0.155	2.532	0.359	1.364	8.478	0.457	10.658
USB	4.55	2.583	0.258	2.841	0.525	1.363	8.217	0.459	10.564
USB	4.60	2.531	0.103	2.634	0.723	1.422	8.383	0.476	11.004
WSB	4.73	2.116	0.258	2.374	1.516	1.362	8.409	0.568	11.856
WSB	4.71	2.168	0.310	2.478	1.351	1.539	8.352	0.576	11.818
WSB	4.91	1.289	0.309	1.599	1.796	1.213	8.521	1.135	12.664
WSB	4.88		0.206		1.846	1.155	8.515	1.137	12.653
WSB	4.71	1.805	0.258	2.063	1.515	1.302	8.414	0.802	12.032
WSB	4.75	1.857	0.258	2.115	1.416	1.184	8.333	0.803	11.736
WSB	4.68	1.755	0.258	2.013	1.632	1.214	8.445	0.859	12.150
WSB	4.72	1.858	0.310	2.168	1.599	1.185	8.480	0.897	12.161
WSB	4.73	1.653	0.258	1.912	1.517	0.980	8.409	1.008	11.914
WSB	4.73	1.705	0.310	2.015	1.137	0.744	8.159	0.959	10.998
WSB	4.61	1.959	0.258	2.217	1.762	1.125	8.178	0.845	11.910
WSB	4.61	2.010	0.258	2.268	1.762	1.184	8.261	0.816	12.023
WSB	4.68	2.241	0.306	2.547	1.529	0.908	8.006	0.721	11.164
WSB	4.71	2.241	0.255	2.496	1.186	0.821	8.179	0.701	10.887
WSB	4.75	1.494	0.258	1.752	1.233	0.889	8.877	0.963	11.962
WSB	4.79	1.443	0.258	1.700	2.074	1.153	8.731	1.006	12.965

[*] Exchangeable acidity = Exchangeable Al^{3+} + Exchangeable H^{+}; Exchangeable bases = Exchangeable K^{+} + Exchangeable Na^{+} + Exchangeable Ca^{2+} + Exchangeable Mg^{2+}.

[**] CK, control; ASB, aboveground soybean parts; USB, underground soybean parts; WSB, whole soybean plants.

S4 Table. Original data of soil NH_4^+, NO_3^-, organic matters and enzyme activities sampled on 12/31/2018

Treatment*	NH_4^+ (mg/kg)	NO_3^- (mg/kg)	Organic Matters (%)	Urease (mg/g/24h)	Sucrose (mg/g/24h)	Nitrate reductase (mg/g/24h)
CK	6.346	6.538	1.466	1.486	3.486	2.585
CK	6.262	6.308	1.522	1.407	3.681	2.261
CK		6.154	1.545	3.032	3.584	3.037
CK	8.815	6.308	1.373	2.853	3.697	3.295
CK	6.515	7.923	0.916	2.952	4.005	3.360
CK	6.308	8.615	0.904	2.833	4.183	3.038
CK	6.262	7.077	1.375	2.378	3.875	2.907
CK	7.177	7.462	1.281	2.516	3.794	2.973
ASB	7.038	5.308	1.891	3.864	4.038	3.553
ASB	5.323	5.538	1.865	3.844	3.876	3.941
ASB	10.085	6.462	1.673	4.359	4.297	3.491
ASB	6.431	6.154	1.487	4.280	4.200	3.617
ASB	4.731	5.154	1.946	3.566	4.054	2.907
ASB	4.462	5.385	2.162	3.685	4.103	3.165
ASB	5.569	5.462	1.775	3.784	4.135	2.648
ASB	5.708	5.846	1.810	3.883	4.037	3.165
ASB	6.900	7.846	1.622	3.170	4.443	4.137
ASB	8.431	7.769	1.844	3.705	4.378	3.425
ASB	4.377	10.231	1.669	3.784	4.232	4.007
ASB	6.954	10.769	1.740	4.636	4.183	3.814
ASB	5.015	6.308	1.493	3.091	4.605	3.488
ASB	5.331	5.615	1.680	3.586	4.686	4.199
ASB	5.246	8.462	1.622	3.547	4.330	3.297
ASB	6.046	9.615	1.857	3.131	4.426	2.780
USB	4.831	5.692	1.446	3.586	3.989	3.618
USB	5.369	6.000	1.432	3.864	3.940	3.103
USB	5.769	9.385	1.752	3.547	4.070	3.166
USB	5.685	9.308	1.621	3.368	4.135	3.554
USB	7.431	8.846	1.727	4.161	4.119	3.295
USB	6.500	8.615	1.714	4.379	4.151	3.360
USB	7.677	10.077	1.751	3.349	3.989	3.231
USB	5.631	11.077	1.740	3.368	4.135	3.295
USB	6.415	7.538	1.646	4.399	4.297	3.876
USB	5.692	7.462	1.539	4.617	4.378	3.749
USB	6.600	7.692	1.688	4.300	4.345	3.360
USB	6.815	9.000	1.836	4.636	4.232	3.814
USB	7.038	4.846	1.631	4.894	4.362	3.619
USB	7.708	5.615	1.749	5.112	4.411	3.878

USB	7.562	12.308	1.702	3.765	4.670	3.684
USB	9.269		1.717	3.705	4.735	3.943
WSB	6.862	8.154	2.104	4.359	4.961	4.006
WSB	6.523	8.077	2.131	4.418	4.849	4.137
WSB	7.608	4.077	2.218	4.557	4.929	4.265
WSB	7.900	4.154	2.286	4.379	4.702	4.331
WSB	11.500	5.923	1.693	4.022	4.540	3.553
WSB	9.738	5.385	1.751	4.042	4.848	4.069
WSB		4.769	1.937	3.408	4.670	3.230
WSB	10.108	4.538	1.951	3.428	4.572	3.361
WSB	6.631	8.769	2.135	4.953	5.076	5.039
WSB	6.938	8.385	2.150	5.231	4.946	4.590
WSB	7.854	9.231	1.919	4.438	5.059	4.781
WSB	9.538	9.000	1.986	4.181	5.108	5.103
WSB	7.677	7.923	2.069	4.676	4.929	4.458
WSB	8.485	8.077	2.015	4.617	4.832	4.327
WSB	8.877	7.385	2.136	5.488	5.124	5.688
WSB	9.823	7.077	2.039	5.429	5.157	5.428

* CK, control; ASB, aboveground soybean parts; USB, underground soybean parts; WSB, whole soybean plants.

S5 Table. Analysis of soil microbial richness[1]

treatment	Clean Tags[2]		OTU[3]		ACE[4]		Chao1[5]	
	Bacterium	Fungus	Bacterium	Fungus	Bacterium	Fungus	Bacterium	Fungus
CK	65847	66042	720	106	845.074	106.543	863.519	106.000
CK	61432	73391	1126	209	1148.320	212.159	1155.714	212.000
CK	59103	72968	1111	197	1129.621	237.536	1144.060	210.750
ASB	61857	70496	1294	273	1323.139	301.801	1333.000	284.769
ASB	60901	51660	1194	210	1261.764	302.997	1259.000	246.111
ASB	61034	69950	1169	191	1259.321	226.340	1261.329	233.750
ASB	61804	71416	1271	183	1300.635	294.612	1303.577	203.000
ASB	60237	70448	1282	259	1318.974	305.138	1336.286	293.000
ASB	59990	68973	1275	212	1308.986	246.871	1329.863	250.000
USB	61499	71579	954	182	1048.682	258.118	1061.342	219.500
USB	62349	64491	1280	189	1321.186	401.380	1328.510	437.000
USB	60160	71129	1224	227	1240.203	283.744	1249.519	266.000
USB	61245	70985	1180	171	1257.046	210.971	1270.470	197.250
USB	60681	58680	1240	151	1295.001	322.289	1310.957	227.500
USB	59398	70685	1193	165	1219.871	213.850	1227.130	192.200
WSB	61752	70821	1195	290	1274.906	306.522	1277.343	317.188
WSB	61657	70891	1201	226	1305.519	259.997	1304.147	253.200
WSB	62426	70757	1187	232	1301.859	238.844	1318.444	243.000
WSB	60799	72004	1220	217	1326.110	270.418	1331.409	260.875
WSB	62331	70952	1278	236	1351.372	253.193	1351.537	249.571
WSB	60763	72461	1321	247	1371.384	250.599	1377.509	250.600

[1]Abbreviations used: CK, control; ASB, aboveground soybean parts; USB, underground soybean parts; WSB, whole soybean plants.

[2] Tags were generated by FLASH, cleaned and filtered by Trimmomatic and UCHIME.

[3]Clean tags with 97% sequence identity was clustered together as an operational taxonomic unit (OTU) using USEARCH.

[4]An index based on abundance-based coverage estimator to measure the richness of the microbiome.

[5] An index based on the number of expected OTUs in a sample among all OTUs identified in all samples, another measure of microbial richness.

S6 Table. ANOVA-Duncan multiple range tests of soil pH for Fig 1.

Treatment*	09/09/2017**	05/07/2018	12/31/2018
CK	4.53±0.01a	4.58±0.02b	4.64±0.02b
ASB	4.54±0.02a	4.65±0.02a	4.72±0.02a
USB	4.55±0.01a	4.64±0.02ab	4.60±0.01b
WSB	4.53±0.02a	4.63±0.02ab	4.73±0.02a

* CK, control; ASB, aboveground soybean parts; USB, underground soybean parts; WSB, whole soybean plants.

** Data represent mean±standard error. Different letters indicate significant differences among treatments at the same time ($P<0.05$).

S7 Table. ANOVA-Duncan multiple range tests of soil NH_4^+ and NO_3^- for Fig 2.

Treatment*	NH_4^+**	NO_3^-
CK	6.81±0.36b	7.05±0.31a
ASB	6.10±0.38b	7.00±0.47a
USB	6.62±0.28b	8.23±0.55a
WSB	8.40±0.38a	6.93±0.46a

* CK, control; ASB, aboveground soybean parts; USB, underground soybean parts; WSB, whole soybean plants.

** Data represent mean±standard error. Different letters indicate significant differences among treatments ($P<0.05$).

S8 Table. ANOVA-Duncan multiple range tests of soil enzymes activities for Fig 3.

Treatment*	Urease**	Sucrase	Nitrate reductase
CK	2.43±0.23c	3.79±0.08c	2.93±0.13c
ASB	3.74±0.11b	4.25±0.05b	3.48±0.12b
USB	4.07±0.14ab	4.25±0.06b	3.53±0.07b
WSB	4.48±0.15a	4.89±0.05a	4.40±0.17a

* CK, control; ASB, aboveground soybean parts; USB, underground soybean parts; WSB, whole soybean plants.

** Data represent mean±standard error. Different letters indicate significant differences among treatments ($P<0.05$).

S9 Table. ANOVA-Duncan multiple range tests of organic matters sampled on 12/31/2018 from S6 Table

Treatment[*]	Organic Matters[**]
CK	1.30 ±0.09%a
ASB	1.76 ±0.04%b
USB	1.67 ±0.03%b
WSB	2.03 ±0.04%c

[*] CK, control; ASB, aboveground soybean parts; USB, underground soybean parts; WSB, whole soybean plants.

[**] Data represent mean±standard error. Different letters indicate significant differences among treatments ($P<0.05$).
